# Initial Clustering Based on the Swarm Intelligence Algorithm for Computing a Data Density Parameter

**DOI:** 10.1155/2022/6408949

**Published:** 2022-06-10

**Authors:** Wei Xiong

**Affiliations:** Jiangxi University of Engineering, Xinyu 338029, China

## Abstract

To improve the accuracy and efficiency of cluster startup using data density parameters, the author proposes a large data cluster extraction algorithm based on a herd intelligence algorithm. Since clustering to initiate data density parameters is primarily data mining, the author explores data mining clustering based primarily on herd intelligence algorithms. First, the obscure c-key cluster algorithm in the clustering algorithm is analyzed, and then the hybrid jump algorithm in the sub-heuristic herd intelligence optimization technology is optimized in the case of a few parameters by combining the obscure c-means cluster algorithm. The simulation results show that the convergence speed of the fuzzy C-means clustering algorithm and hybrid leapfrog algorithm is slow; the convergence rate of the PSO-FCM algorithm has been improved. Since the fusion algorithm requires fewer adjustment parameters, the cluster centers can be obtained more accurately and quickly with strong robustness and fast convergence. Compared with other algorithms, the fusion algorithm proposed by the author has the best performance in clustering effect, accuracy, convergence rate, and robustness. It is proved that the swarm intelligence algorithm can effectively perform density parameter initialization clustering on computational data.

## 1. Introduction

Clustering is a common technology for extracting data, and cluster algorithms are widely used in many fields, such as pattern recognition, machine learning, image processing, and information retrieval, and play an important role in data mining. It can divide a large amount of data into several categories or clusters in a similar way. The similarity of the data in a cluster is relatively high, while the similarity of the data in different clusters is relatively small. Many scientists have done in-depth research on clusters and proposed a variety of cluster algorithms, such as hierarchical clusters, split clusters, and density-based clusters. The continuous development of information technology has created enormous amounts of data and made it increasingly difficult for people to process the data. The use of data mining technology is an effective tool for extracting valuable data from large amounts of data. The data mining algorithms are gradually becoming a hotspot of research that people are interested in. Cluster analysis is one of the most important areas of data mining, and classifying data by collecting similar data is one of the main tasks of a data mining algorithm [1]. However, as the number of iterations increases, traditional k-means and fuzzy-based algorithms are less efficient, making it difficult to guarantee data mining quality. In response to this situation, a large data cluster mining technology was developed based on cloud agglomeration intelligence algorithms. Today's networking is huge, with many data processing options available in the cloud, which use cloud computing to integrate multiple computer resources. It allows users to overcome time and space constraints and flexibly access unlimited resources and algorithms in the cloud environment. Swarm intelligence algorithms are emerging evolutionary computational technologies closely related to artificial intelligence, and include mainly ant colony algorithms and particle herd algorithms [2–4].

## 2. Related Works

Deep, V. intelligent search systems based on particle herd algorithms will be studied, and particle herd algorithms will be introduced into intelligent search engines to implement relevant searches for large public safety information [5]. The intelligent group cluster approach of group cooperatives adopted by Kim SS introduced a multigroup evolution scheme in the particle herd algorithm, which avoids local optimization solutions, improves the efficiency, accuracy, and data storage performance of information cluster. However, there are still some limitations that do not fit the cloud computing model, such as FOPTICS algorithm [6]. Jabbar, A. M. and others improved the DBSCAN (density-based spatial clustering of applications with noise) algorithm for uncertain data, the FDBSCAN (fuzzy DBSCAN) algorithm is proposed; the FDBSCAN algorithm adopts a similar improvement to FOPTICS [7]. Kaur, K. and other scholars studied the probabilistic properties of uncertain data and further improved the density-based FDBSCAN algorithm combined with the probability index; at the same time, the selection method of the initial parameters is optimized, and the P-DBSCAN (probabilistic DBSCAN) algorithm is proposed [8]. Khodadadi, E. and other scholars have studied the K-means algorithm and the probability distribution and probability density of uncertain data, it is proved that the uncertainty data can be represented using its probability density function, at the same time, minimize the expected squared errors *E* (SSE) as the similarity measure between objects and clusters, then a classical partition-based uncertainty data clustering algorithm UK-means (uncertain K-means, UK-means) algorithm is proposed. However, when the cluster center of this algorithm is updated, the expected distance needs to be calculated again, and the calculation amount is too large [9]. Therefore, Usman, D. and other scholars aimed at these deficiencies, the similarity measurement method between data objects is improved, so that the amount of calculation can be reduced, save time overhead, and then propose CK-means (Classical K-Means, CK-Means) algorithm [10]. Liu, J. and other scholars proposed the UID-DBSCAN (uncertain multidimension data DBSCAN, UID-DBSCAN) algorithm; compared with the above algorithms, this algorithm is fundamentally different in the way of improvement, the algorithm effectively combines interval numbers and statistical values in the process of describing uncertain data, in the process of distance calculation, the distance calculation method between interval numbers with low complexity is adopted. Therefore, the clustering effect of the algorithm has been significantly improved. However, this algorithm is sensitive to initial parameters and cannot identify clusters of arbitrary density [11]. Wiharto, W. and other scholars improved the PAM (partitioning around medoid, PAM) algorithm by using the interval number combined with the standard deviation of the data, proposed U-PAM (uncertain partitioning around medoid, U-PAM) algorithm, UM-PAM algorithm, and in order to determine the optimal number of clusters to make the clustering accuracy better, the algorithm introduces the CH index to provide guarantee, in order to a certain extent, the clustering accuracy has been improved. However, they still have the disadvantage of not being able to find clusters of arbitrary shapes [12]. Smith, A. J. and other scholars proposed the IUK-means algorithm, which is an uncertain data clustering algorithm based on fast Gaussian transformation; in the similarity measurement, the attribute characteristics of the uncertainty data are combined with their probability density functions, the similarity between objects is more accurately measured to complete the clustering. Clustering algorithms for uncertain data streams and high-dimensional uncertain data are also a research hotspot. Continuity, real-time, high speed, etc. are the characteristics of uncertain data flow. The application of data stream processing is very extensive, and its real-time requirements are high, the amount of data is also huge. Therefore, the research on cluster mining of uncertain data streams is also a difficult problem that researchers are waiting to solve. In addition, the uncertainty of the data will have an impact on high-dimensional data, this makes high-dimensional data more sparse, and the problem of how to deal with high-dimensional uncertain data needs to be solved urgently [13]. For the problem of uncertain data flow, scholars such as C González-Santos proposed the UMicro algorithm, this algorithm introduces the concept of microclusters in deterministic data flow and deeply studies the CF structure, it is extended to deal with the uncertainty of the data, and the radii of each cluster and the similarity measure of the object to the cluster are improved [14].

Based on current research, this paper proposes a large data cluster mining algorithm based on herd intelligence algorithms. Since clustering to initiate data density parameters is primarily data mining, this manuscript examines data mining clustering based primarily on herd intelligence algorithms. First, the obscure C grouping algorithm in the clustering algorithm was analyzed, and then the hybrid frog jump algorithm in the subheuristic herd intelligence optimization was combined with the obscure C-key cluster to optimize global search capability to suit low parameter conditions.

## 3. Cluster Analysis

### 3.1. Related Concepts of Cluster Analysis

#### 3.1.1. Initialize the Cluster Center

Using the ant colony algorithm in the majestic intelligence algorithm, the cluster center assumes that the ant is the food source, and the entire data package is the ant-seeking and agglomeration process. Looking for a food source. Suppose there is a data set *Q*={*Q|q*_*i*1_, *q*_*i*1_,…, *q*_*in*_}, *i*=1,2,…, *m*. Here, *N* and *m* are constant, *λ* is the pheromone size, and *l* (*I*, *O*) is the distance from the *I* data to the center of the *O* cluster. Adjust the pheromone between all data to a certain constant *C*, adjust the pheromone update formula between different data objects, combine the data objects into cluster centers through the update formula, and create a data set that is aggregated in the *Q* field. The special calculation formula is as follows:

(1) $$\begin{array}{c} \left\{\begin{array}{c} \Delta \lambda_{ij}=\frac{A}{l\left(i,j\right)+B},\\ \lambda_{ij}\left(t+1\right)=k\lambda_{ij}\left(t\right)+\Delta \lambda_{ij}, \end{array}\right) \end{array}$$

(2) $$\begin{array}{c} C_{j}=\frac{1}{N}\sum_{i=1}^{N}Q_{i},\;Q_{i}\in C_{j}. \end{array}$$

Formula (1) is the pheromone update formula, and formula (2) is the merge formula, where, *A* and *B* represent normal numbers, *k* represents residual pheromone intensity, *t* represents time, *C*_*j*_ represents the merged data set, *N* represents the number of data in the data set, and *λ*_*ij*_(*t*) represents the size of the pheromone between data *i* and *j* at time *t*. Through the above process to complete the initialization of the cluster center, the process of cluster center initialization also represents the initial iterative process.

#### 3.1.2. Calculate the Data Density Parameter and the Distance between Classes

In an ant colony algorithm, the underlying data are usually randomly selected, which leads to an uneven distribution of data, which affects the overall performance of the algorithm [10]. Therefore, cluster centers are updated using density-based and max-min distance methods. Calculate the distance between any two data, record it in a matrix, get $\bar{l}$ between the two data, then according to the Density(*q*_*i*_) ≤ Density(*q*_*i*_)/4 principle, the isolated samples are excluded from the data set *C*_*j*_, in order to get the updated cluster center, the formula is as follows:

(3) $$\begin{array}{c} \left\{\begin{array}{c} \bar{l}=\frac{\sum l\left(i,j\right)}{n^{2}},\\ \text{Density}\left(q_{i}\right)=\left\{e\in C_{j}|l\left(q_{i},e\right)\leq r\right\}, \end{array}\right) \end{array}$$

where $r=\lambda \times \bar{l};\text{Density}\left(q_{i}\right)$ represents the density of *q*_*i*_. After completing the update of the cluster centers, for other data samples in the data set *C*_*j*_, the distance from its center to the updated cluster center is calculated through the matrix, and the number of calculations is the same as the number of iterations. By calculating the data density parameter and the distance between classes, the cluster center upgrade is completed and the optimal solution with the least space is obtained, and the solution in this case is optimal partition clustering [15].

As can be seen from the introduction above, the nature of cluster analysis is a matter of multidimensional sampling and classification. To do this, the concept of “space” was introduced. Assuming that *X*={*x*_1_, *x*_2_,…, *x*_*p*_} and *Y*={*y*_1_, *y*_2_,…, *y*_*p*_} are two sample data of size *p*, the method for calculating the absolute distance is given in (4).

(4) $$\begin{array}{c} d\left(X,Y\right)=\sum_{i=1}^{p}\left|x_{i}-y_{i}\right|. \end{array}$$

In addition, there are calculation methods such as the Chebyshev distance, Mahalanobis distance, and Euclidean distance.

(5) $$\begin{array}{c} q_{ij}=\frac{\sum_{k=1}^{p}x_{ik}x_{jk}}{\sqrt{\sum_{k=1}^{p}x_{ik}^{2}\sum_{k=1}^{p}x_{jk}^{2}}}. \end{array}$$

Uncertain cluster methods are often used to solve uncertain and uncertain problems, such as weather forecasts. In an uncertain cluster analysis data model, the initial data matrix is defined as follows.

(6) $$\begin{array}{c} \left(\begin{array}{cccc} x_{11} & x_{12} & \ldots & x_{1n}\\ x_{21} & x_{22} & \ldots & x_{2n}\\ \ldots & \ldots & \ldots & \ldots \\ x_{n1} & x_{n2} & \ldots & x_{mn} \end{array}\right). \end{array}$$

The fuzzy C-clustering algorithm belongs to the objective function-based method and shows good performance when working with large amounts of data. Therefore, the object of this study is the little-known C cluster algorithm [16].

### 3.2. Fuzzy C-Means Clustering Algorithm

The fuzzy C-means clustering algorithm adopts the idea of hard clustering. Let *X*={*x*_*i*_, *x*_2_,…, *x*_*n*_} ⊂ *R*^*s*^ denote a dataset, *x*_*j*_={*x*_*l*1_, *x*_*j*2_,…, *x*_*jk*_,…, *x*_*jn*_} ⊂ *R*^*s*^ represents the *s* eigenvectors of the *j* th data sample, and *x*_*jk*_ represents the feature value of the *j* th data sample on dimension *k*. The dataset *X* is grouped into C subsets *Y*={*X*_1_, *X*_2_, ..., *X*_*C*_}, *C* ∈ [2, *n*]. If *Y* meets the requirements of (7), then *Y* can be regarded as a hard *C* group.

(7) $$\begin{array}{c} \left\{\begin{array}{c} X_{1}\cup X_{2}\cup \cdots \cup X_{C}=X,\\ X_{i}\cup X_{K}=\varphi ,1\leq i\neq k\leq C,\\ X_{i}\neq \varphi ,X_{i}\neq X,1\leq i\leq C. \end{array}\right) \end{array}$$

Let the membership degree of sample *x*_*j*_ belonging to subset *X*_*i*_ be *u*_*ij*_, then the membership matrix *U*=[*μ*_*ij*_]_*C*×*n*_ can be used to represent the hard C grouping, among them *μ*_*ij*_ ∈ {0,1}. Therefore, the hard-C grouping of dataset *X* can be represented by

(8) $$\begin{array}{c} M_{fc}=\left\{U\in R^{s}|u_{ki}\in \left\{0,1\right\};\sum_{i=1}^{C}u_{ki}=1,\forall k;0<\sum_{k=1}^{n}u_{ki}<n,\forall i\right\}. \end{array}$$

Therefore, the fuzzy *C* grouping can be represented by

(9) $$\begin{array}{c} M_{fc}=\left\{U\in R^{s}|u_{ki}\in \left[o,1\right],\forall i,j;\sum_{i=1}^{C}u_{ki}=1,\forall k;0<\sum_{k=1}^{n}u_{ki}<n,\forall i\right\}. \end{array}$$

For dataset *X*={*x*_1_, *x*_2_,…, *x*_*k*_,…, *x*_*n*_} ⊂ *R*^*s*^, *n* is the number of elements in dataset *X*, and *s* is the number of attribute values in sample *x*_*k*_. *c* cluster centers form a matrix *V*={*v*_1_, *v*_2_,…,*v*_*i*_,…,*v*_*c*_}_*S*×*C*_, where *v*_*i*_={*v*_*i*1_, *v*_*i*2_, *v*_*is*_} is the element of the *i*-th cluster center, *c* is the number of categories of clusters, then the objective function of the fuzzy *C*-means clustering algorithm is as follows:

(10) $$\begin{array}{c} J_{m}\left(U,V\right)=\sum_{i=1}^{C}\sum_{j=1}^{n}u_{ij}^{m}\cdot d_{ij}^{2}. \end{array}$$

The constraints are as follows:

(11) $$\begin{array}{c} \left\{\begin{array}{c} \sum_{i=1}^{c}u_{ij}=11\leq j\leq n,\\ 0\leq u_{ij}\leq 11\leq i\leq c,1\leq j\leq n,\\ 0\leq \sum_{j=1}^{n}u_{ij}\leq n1\leq i\leq c. \end{array}\right) \end{array}$$

The Euclidean distance from the *j* th sample data *x*_*j*_ to the *i*-th cluster center *v*_*i*_ is as follows:

(12) $$\begin{array}{c} d_{ij}=\left\|x_{j}-v_{i}\right\|. \end{array}$$

The calculation methods of the *i*-th cluster center *v*_*i*_ and membership degree *u*_*ij*_ are shown in equations (13) and (14), respectively.

(13) $$\begin{array}{c} v_{i}=\frac{\sum_{j=1}^{n}u_{ij}^{m}\cdot x_{j}}{\sum_{j=1}^{n}u_{ij}^{m}},\;j=1,\ldots ,c, \end{array}$$

(14) $$\begin{array}{c} u_{ij}=\frac{1}{\sum_{k=1}^{c}{\left(d_{ij}/d_{ik}\right)}^{2/m-1}},\;i=1,\ldots ,n;\;j=1,\ldots ,c. \end{array}$$

### 3.3. Fusion of the Hybrid Leapfrog Algorithm and Fuzzy C-Means Clustering Algorithm

#### 3.3.1. Hybrid Leapfrog Algorithm

**Figure 1:**
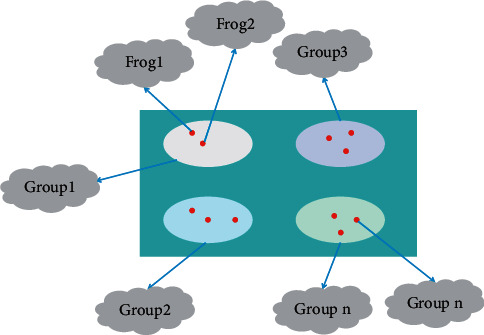
Schematic diagram of the hybrid frog leaping algorithm.

The hybrid leapfrog algorithm can solve the local search problem, and also consider global information [17]. In limited space, the hybrid leapfrog algorithm is performed by simulating the jumping foraging behavior of frog population, in order to solve the combinatorial optimization problem. The algorithm requires fewer adjustment parameters and has strong global search ability, the principle of hybrid leapfrog algorithm is shown in Figure 1.

In a finite space, randomly generated F solutions {*x*^1^, *x*^2^,…, *x*^*F*^} represent the initial population. *x*^*i*^={*x*_1_^*i*^, *x*_2_^*i*^,…, *x*_*s*_^*i*^}(*i*=1,2,…, *F*) represents 1 solution in the *S*-dimensional solution space, where *S* represents the number of information elements each frog has. *f*(*x*^*i*^)(*i*=1,2,…, *F*) represents the fitness of each frog in the population, and sorts all frogs in descending order according to their numerical size. *x*^*g*^ represents the individual with the optimal solution in the population.

The process of the hybrid leapfrog algorithm is as follows: ① Initialization of each parameter of the algorithm; ② division of subpopulations; ③ local search evolution within the group; and ④ mixing of subpopulations. In each iterative step of the local search, only the worst solution in the sub-population needs to be updated once, and the specific method is as follows:

(15) $$\begin{array}{c} x^{w}=x^{w}+D_{i},-D_{\max}\leq D_{i}\leq D_{\max},\;i=1,2,\ldots ,m,\\ D_{i}=\text{rand}\left(\;\right)\cdot \left(x^{b}-x^{w}\right),\;i=1,2,\ldots ,m. \end{array}$$

Among, rand represents a random number greater than 0 and less than 1, *D*_*i*_ represents the distance of each jump of the frog, *D*max represents the maximum distance of each jump of the frog, *x*^*b*^ represents the optimal solution of fitness value in each sub-population, and *x*^*w*^ represents the worst solution of fitness value in each subpopulation [18].

#### 3.3.2. Algorithm Fusion Ideas and Steps

The steps for an obscure *C*-environment clustering algorithm based on a hybrid frog jump are as follows.

**Step 1 .**

Initialization: Initialize the number *N* of frog populations, the number of clusters *c*, and set a random original membership matrix, as the initial clustering division, the cluster centers of each type are calculated according to formula (15), which is used as the frog mapping code in the initial hybrid frog leaping algorithm.

**Step 2 .**

Behavior selection: Calculate the fitness function value of each frog obtained after simulating the frog group behavior according to formula (17), and arrange and group the frogs in descending order.

(16) $$\begin{array}{c} f=\frac{q}{J_{m}+1}, \end{array}$$

where *q* is a random number whose value range is [0, 1] and *J*_*m*_ represents the objective function of fuzzy C-means clustering (equation (9)).

**Step 3 .**

Do a local search on each subpopulation and update the worst person in the subpopulation until the local search iteration peaks.

**Step 4 .**

Mix subpopulations and update optimal population solutions in a timely manner.

**Step 5 .**

Repeat steps 2-5 until the maximum number of iterations is given, at which point the global optimal solution becomes the first center of the cluster. Invoke a fuzzy C-application cluster algorithm to obtain the final cluster matrix grouping. Finally, the maximum membership rule is used to label all samples in the data set [19].

**Figure 2:**
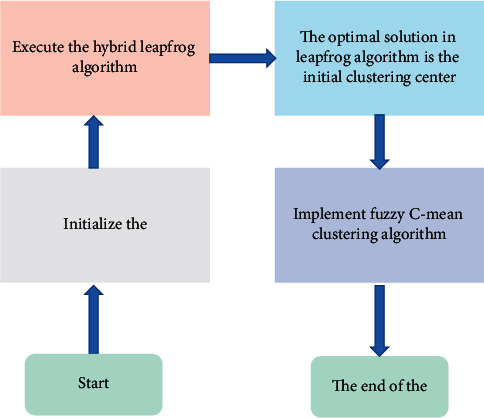
Flow of the fusion algorithm.

The flow of the fusion algorithm is shown in Figure 2.

## 4. Simulation Results

### 4.1. Experimental Dataset

To test the effectiveness and progress of the aggregation algorithm proposed in this article, the traditional fuzzy C-note clustering algorithm, the hybrid frog jump algorithm, the PSO-FCM algorithm, and the algorithm described in this article were all performed using conventional Iris. Dataset and three artificial data sets Dataset 1. Simulation. The parameters of the four test data sets are shown in Table 1.

### 4.2. Clustering Effect Analysis

In the experiment, the maximum number of iterations was 500, the number of subclusters was 5, the number of subclusters was 3, the population size was 30, the indeterminate index was *m* = 2, and the number of iterations in the array was 5. The four algorithms were repeated 40 times to calculate the average value of each parameter. The test results for the four algorithms on different data sets are shown in Tables 2–5. The accuracy of the cluster results is assessed by the accuracy of the classification, and the method for calculating the accuracy of the classification is shown in (17) [20].

(17) $$\begin{array}{c} T=\frac{M}{N}\times 100\%, \end{array}$$

where *M* represents the correct number of sample clusters and *N* represents the total number of data objects contained in the dataset.

Table 2 shows that the cluster effect of the dim *C*-key cluster algorithm is relatively poor because it is more sensitive to the initial value. Although the hybrid frog jumping algorithm is well robust and has a unifying speed, it overcomes local overvaluation problems but lacks accuracy when dealing with large data cluster problems. The PSO-FCM algorithm combines a particle optimization algorithm with a dim *C*-notification cluster algorithm to take advantage of mass intelligence optimization to improve local search. We optimize cluster effects to gain capabilities and greater accuracy. The optimal solution for the author's proposed aggregation algorithm is to adjust the center value of the cluster in a faint *C* cluster, select the fitness function optimally, improve global search capability and search accuracy, and achieve good results. The cluster effect is similar to the PSO-FCM algorithm.

### 4.3. Comparison of Convergence Speeds

**Figure 3:**
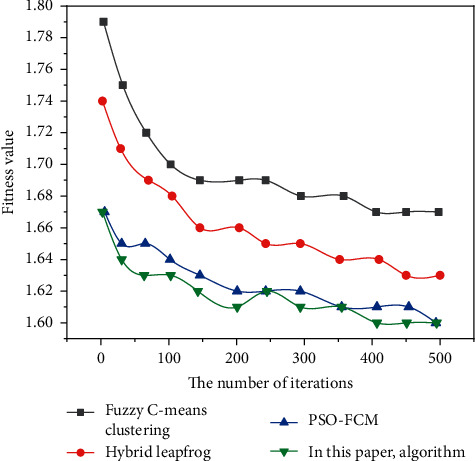
Test results on Dataset 1.

**Figure 4:**
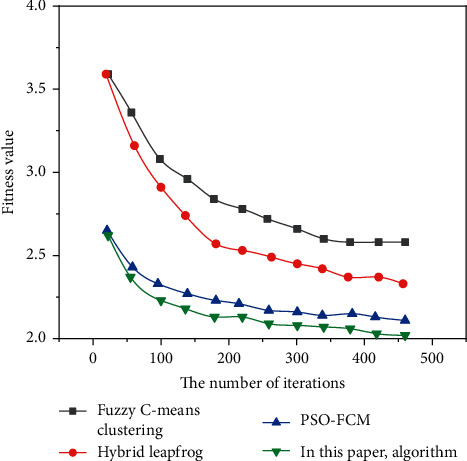
Test results on the Dataset 2.

**Figure 5:**
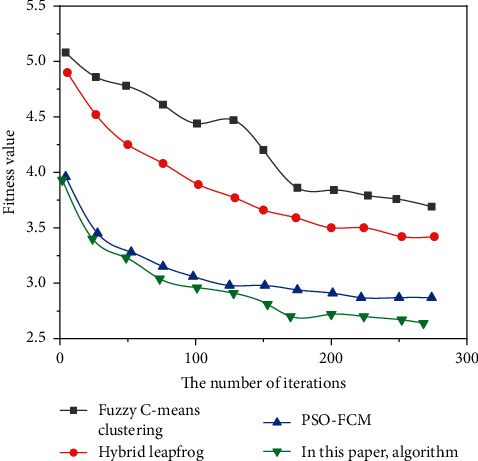
Test results on the Dataset 3.

Figures 3–5 show the comparison results of the convergence rates obtained by applying the traditional fuzzy C-means clustering algorithm, the hybrid frog leaping algorithm, the PSO-FCM algorithm, and the author's algorithm to Dataset 1, Dataset 2, and Dataset 3, respectively.

As can be seen from Figures 3to 5, the convergence rate of the fuzzy C-means clustering algorithm and hybrid frog leaping algorithm is slow. The convergence rate of the PSO-FCM algorithm is improved. Since the fusion algorithm requires fewer adjustment parameters, it can obtain the cluster centers more accurately and quickly, with strong robustness and fast convergence [21].

Compared with several other algorithms, the fusion algorithm proposed by the author has the best performance in clustering effect, accuracy, convergence rate, and robustness.

## 5. Conclusion

The test shows that the improved algorithm proposed in this paper has good clustering effect, high accuracy, fast convergence speed, and good comprehensive performance. It can be applied to data mining and clustering. With the rapid development of networking and information technology, data are becoming more valuable and at the same time, more and more valuable. to. Combining herd intelligence algorithms and cluster analysis algorithms with the development of large data cluster mining technologies in the cloud environment has the clear advantage of solving the problems of traditional data cluster mining and increasing the efficiency along with the algorithm. Real value. However, due to the limited experimental environment, it is necessary to manually adjust the starting constant of the herd intelligence algorithm, and there are some deviations. Further research and discussion is needed on how to solve this problem and obtain the optimal constant.

## Figures and Tables

**Table 1:** Parameters of 4 experimental datasets.

data set	Number of categories	Number of samples	number of attributes
Iris	3	150	4
Dataset 1	5	400	3
Dataset 2	8	500	6
Dataset 3	3	200	5

**Table 2:** Experimental results on the Iris dataset.

algorithm	Total	The correct number of sample clusters	Correct rate/%
Fuzzy C-means clustering	150	138	92
Hybrid leapfrog	150	132	88
PSO-FCM	150	129	86
This article	150	139	93

**Table 3:** Experimental results on Dataset 1.

algorithm	Total	The correct number of sample clusters	Correct rate%
Fuzzy C-means clustering	400	351	88
Hybrid leapfrog	400	339	85
PSO-FCM	400	318	79.5
This article	400	333	83

**Table 4:** Experimental results on Dataset 2.

algorithm	Total	The correct number of sample clusters	Correct rate%
Fuzzy C-means clustering	500	367	73
Hybrid leapfrog	500	379	76
PSO-FCM	500	312	62
This article	500	309	61.8

**Table 5:** Experimental results on Dataset 3.

algorithm	Total	The correct number of sample clusters	Correct rate/%
Fuzzy C-means clustering	200	129	64
Hybrid leapfrog	200	135	67
PSO-FCM	200	150	7572
This article	200	145	

## Data Availability

The data used to support the findings of this study are included within the article.
